# Non-traditional students’ preferences for learning technologies and impacts on academic self-efficacy

**DOI:** 10.1007/s12528-023-09354-5

**Published:** 2023-01-24

**Authors:** Karen Sutherland, Ginna Brock, Margarietha. J. de Villiers Scheepers, Prudence M. Millear, Sherelle Norman, Tim Strohfeldt, Terri Downer, Nicole Masters, Alison. L. Black

**Affiliations:** University of the Sunshine Coast, Sippy Downs, Queensland Australia

**Keywords:** Self-efficacy, Academic achievement, Self-regulation, Blended learning, Non-traditional students

## Abstract

Blended Learning (BL) as a pedagogical approach has increased in significance during the COVID-19 pandemic, with blended and online learning environments becoming the new digital norm for higher educational institutions around the globe. While BL has been discussed in the literature for thirty years, a common approach has been to categorise learner cohorts to support educators in better understanding students’ relationships with learning technologies. This approach, largely unsupported by empirical evidence, has failed to adequately address the challenges of integrating learning technologies to fit with non-traditional students’ preferences, their BL self-efficacy and the associated pedagogical implications. Focusing on student preference, our study presents findings from a pre-COVID survey of undergraduate students across four campuses of an Australian regional university where students shared their learning technology preferences and the self-regulated learning that influenced their academic self-efficacy in a BL context. Findings show students want consistency, relevance, and effectiveness with the use of BL tools, with a preference for lecture recordings and video resources to support their learning, while email and Facebook Messenger were preferred for communicating with peers and academic staff. Our study suggests a quality BL environment facilitates self-regulated learning using fit-for-purpose technological applications. Academic self-efficacy for BL can increase when students perceive the educational technologies used by their institution are sufficient for their learning needs.

## Introduction

Blended Learning (BL) has become a topic of great importance since the emergence of COVID-19 (Moszkowicz et al., 2020). In response to this global pandemic, higher education institutions have been forced to rapidly embrace BL and make use of a host of technology tools to maintain their educational offerings to students (Crawford et al., 2020), without the time or a clear understanding of what tools students consider beneficial to their learning. Previous research has attempted to illuminate students’ relationships with technology by assigning learners to categories such as the *Net Generation* (Tapscott, 1998), *Net Gen* (Howe & Strauss, 2000), and *Digital Natives* (Prensky, 2001). These categorisations suggest students from younger age demographics possess innate technological fluency, which empower them with enhanced learning and operational efficiencies (Prensky, 2001). Subsequent critique emphasises a lack of empirical support for these predictions (Bennett et al., 2008).

Complicating the issue is that universities are increasingly teaching diverse, non-traditional student cohorts (Meuleman et al., 2015). Students from non-traditional backgrounds are diverse in their social, family and education background, tend to have previous work experience, are older (23 or 25 years at their time of enrolment) and their life situation and motivation to study differs from traditional school leavers (Gilardi & Guglielmetti, 2011). These student cohorts are less familiar with university BL expectations and face transition challenges as many are first-in-family (Meuleman et al., 2015), which creates pedagogical challenges for educators in designing meaningful BL experiences.

Non-traditional students face barriers in acquiring and using digital technologies (Kuo & Belland, 2019). For example, in the US African Americans are less likely to go online and have access to broadband services at home, compared to traditional students (Kuo & Belland, 2019). The Australian Digital Inclusion index shows that non-traditional students in regional and rural geographies often experience affordability and access barriers as online learning activities demand higher data allowances (Thomas et al., 2020). Yet limited research has focused on non-traditional students’ technology preferences, their BL self-efficacy and the implications of integrating learning technologies to fit with students’ preferences. These factors hold important implications for designing BL experiences conducive to achieving learning outcomes and developing students’ self-efficacy.

Given this research gap, the aim of this study is to determine how non-traditional students’ learning technologies preferences and their self-regulation influence their self-efficacy for blended learning and what the pedagogical implications are for designing BL experiences. Specifically, we ask: (i) what technological tools do non-traditional students use to support their learning? (ii) what predictors influence non-traditional students’ self-efficacy and perceived confidence to complete their studies, and (iii) what are the pedagogical implications for designing BL experiences? Using a survey design, we explore which technologies non-traditional students prefer for learning, ascertain how differences in age, gender and first-in-family and self-regulation relates to non-traditional students’ academic self-efficacy and perceived confidence to complete their unit and degree using hierarchical regression analysis and logistical regression and then draw out pedagogical implications, thereby contributing to the field of non-traditional students’ self-efficacy, their technology preferences for learning and the importance of developing self-regulation during their studies.

## Literature review

Non-traditional students’ learning is influenced by personal factors (demographic characteristics, cognitive perceptions, preferences), environmental factors (social and BL learning environment) and behavioral factors according to social cognitive theory (Bandura, 1997). In the literature review we explicate the personal demographic factors, identified in previous studies, likely to influence non-traditional students’ learning experiences at university, and explore their attitudes towards technology likely to have a bearing on their preferences. We then consider self-regulated learning and its relevance for non-traditional students’ proactive use of cognitive, metacognitive and motivational strategies for learning. Personal demographic factors and cognitive factors are expected to predict academic self-efficacy expectations related to their confidence and mastery to use technologies to assist them in learning. Finally, we consider the implications for BL pedagogy.

### Non-traditional students’ experiences and expectations of using learning technologies

Learning backgrounds, demography and academic skills influence how BL environments are experienced (Pavlakou & Sharpe, 2014). Some studies show students prefer blended courses over traditional delivery (Brooks, 2016), where delivery of course material combines face-to-face and online learning environments (Zhang et al., 2018). This combination can provide richer experiences overall (Van Doorn & Van Doorn, 2014). BL promises more flexibility for non-traditional students who balance family and life responsibilities with their studies (Eversole, 2021). Non-traditional students are a diverse group of students identified through demographic characteristics like age, gender, first-in-family and are located in regional and rural geographies (Eversole, 2021). Mature-aged students, many of whom are women, tend to be the first in their family to go to university and grew up in low-income families, so tend to face substantial socio-cultural and digital access barriers (Gilardi & Guglielmetti, 2011; Meuleman et al., 2015; Thomas et al., 2020). Therefore, students’ demographic characteristics are useful when considering how to design BL experiences likely to influence students’ self-efficacy and confidence to succeed (Brooks, 2016; O’Shea et al., 2015; Zhang et al., 2018).

Non-traditional students are often first in family and unfamiliar with university expectations. An international research project found that these students are less likely to perform well academically (NCES, 2018; Stone et al., 2016), yet Nelson et al., (2017) found low SES students on regional campuses graduate at a comparable rate with high SES students. This discrepancy in academic achievement could be explained by considering that novice adult learners differ from more experienced students, as Yoo & Huang (2013) found that novice adult learners were often overwhelmed by online learning. Ilgaz and Gülbahar’s (2015) found that a lack of interaction with educators and other students, along with technical issues, was problematic for first-in-family students. These challenges could be exacerbated when considering digital access and affordability issues (Thomas et al., 2020), however, if these are addressed, BL is likely to be preferable for this cohort. Specifically, Brooks (2016) found that female and first-in-family students are significantly more likely to have increased levels of engagement and enrichment using BL technology. Online and blended learning enable students to combine study with paid work and family responsibilities (O’Shea et al., 2015). Well-designed BL environments where online learning tools are used to assist students to understand digital technologies and connect with peers, increase student satisfaction with online and blended learning (Lambrinidis, 2014).

Gender can influence the adoption of technology (Zhang et al., 2018). In the Australian population women have lower levels of digital inclusion than men related to digital access, affordability and digital abilities which consider attitudes, basic skills and activities (Thomas et al., 2020). Nevertheless Brooks (2016) report women students performed better than men in online environments, while men preferred face-to-face educational environments (Latchem, 2014; Lederman, 2013; Zhang et al., 2018). Educators need to be aware of these differences and consider ways to alleviate these discrepancies.

Online and blended learning offer students, particularly the mature aged cohort with additional family and work responsibilities, the opportunity to study at university. Age is a contributing factor in students’ learning experience and expectations, as mature aged students might find it hard to adjust to university life in their first year (Meuleman, 2015), yet as they become more experienced at learning they develop their self-efficacy beliefs (Talsma et al., 2018).

Prior academic achievement and performance show a positive relationship with academic self-efficacy, that is students’ beliefs that they will be able to master their coursework and perceptions of their ability to succeed (Honicke & Broadbent, 2016; Hwang et al., 2016) examined the relationship between students’ past academic performance at school, self-efficacy beliefs and academic achievement at university. Findings indicated “the effect of past academic performance on self-efficacy beliefs was larger than the effect of self-efficacy beliefs on academic achievement” (p. 95). Similarly, Komarraju & Nadler (2013) found comparable results in a study examining the relationship between self-efficacy beliefs and academic achievement. Non-traditional students face social, cultural and academic barriers to university studies, as they do not follow the traditional route of academic achievement at school to gain entry to university. Many universities address these challenges by offering enabling programmes to facilitate entry into university for non-traditional cohorts and provide ongoing support (Eversole, 2021). The experiences and prior knowledge of non-traditional cohorts should be considered when tailoring BL pedagogies to address students’ self-efficacy and self-regulated learning.

### Self-regulated learning

A vital factor in students’ learning performance is their ability to take control of their own learning through self-regulation. Self-regulation refers to a proactive process where learners define use motivational, cognitive and metacognitive strategies in their learning to achieve performance goals and is influenced by the context features of the environment (Jung et al., 2021; Pintrich & Zusho, 2002). The contextual features of online and blended learning environments require students to draw on self-regulated learning strategies to perform well. The motivational, cognitive and metacognitive strategies students employ to self-regulate their learning influence their academic self-efficacy and outcomes (Richardson et al., 2012). Student motivation (both intrinsic and extrinsic) is a key part of this ability to perform (Law et al., 2019). Motivation for engagement with BL arises from intrinsic motivation and leads to more positive experiences when compared to a student relying on external reward or extrinsic motivation (Prasad et al., 2018).

Self-regulation is demonstrated in the conscious scrutinizing of steps in the learning process: setting personal learning goals, devising ways to achieve these goals, accessing and managing learning resources, energy invested, seeking and responding to feedback and the artefacts created (Nicol & MacFarlane-Dick, 2006). In a BL context this means students need reference points such as a performance standard against which they can compare their progress. Self-regulation of learning involves cognitions, motivations and goals students use to fulfil their academic requirements (Nicol & MacFarlane-Dick, 2006). Low self-efficacy increases the likelihood individuals will abandon their goals, reduce their efforts, or settle for a lower result (Bandura, 1997). The diversity of student populations makes it critical to consider the different ways learning technologies are experienced.

### Academic self-efficacy

Academic self-efficacy (ASE) refers to an individual’s perception of their capacity to organize, enact academic practices and achieve the confidence and mastery required to achieve specific outcomes (Bandura, 1997). ASE is positively related to academic success (Talsma et al., 2018). Through its impact on self-regulation, self-efficacy reduces procrastination amongst university students (Klassen et al., 2008). Generally, students with higher ASE increase their efforts, persist longer, and tend to engage more deeply with learning tasks which leads to better performance (Kuo & Belland, 2019). ASE provides a foundation for self-assessed intelligence and abilities (Peterson & Whiteman, 2007). Mastery experiences, vicarious experiences, social persuasion and affective states have been shown to be important for the development of students’ self-efficacy (Van Dinther et al., 2011).

Kuo and Belland (2019) argue that self-efficacy for university students should also consider how students judge their ability to perform learning tasks using technological tools and the internet, as these influence their behaviour in a BL contexts. For non-traditional students this means that their self-efficacy in a BL context should take into account their attitudes, potential anxieties, learning process and learning outcomes. An increasing number of studies have identified students’ engagement with and preferences for BL environments is related to self-efficacy (Pellas, 2014). Greater e-learning self-efficacy increases the perceived usefulness and ease of BL technologies and positively influences intentions to use technologies for learning.

### Blended learning pedagogy

The concept of BL – using digital technologies integrated with face-to-face teaching – has been studied for nearly thirty years (Nystrand & Gamoran, 1991). Researchers have debated the idiosyncrasies of BL pedagogies such as the cognitive advantages (Garrison & Kanuka, 2004; Nystrand & Gamoran, 1991), flexibility and diversification offered (Horn & Staker, 2015; Means at al., 2013), technological advantages and disadvantages (Azevedo & Bernard, 1995; Picciano, 2009; Shute, 2008), and the necessity to rethink curriculum (Halverson & Graham, 2019; Vaughan et al., 2013). However, all higher education courses “incorporate information and communications technology to some degree” (Vaughan et al., 2013, p.7). Therefore, the focus needs to shift to explore integration levels, the purpose of digital delivery, and the impact on learner engagement.

Digital technologies are well-established in university learning and teaching programs, with students using word processing applications, email, social media, and internet searches (Bennett et al., 2008). University students often have limited agency with educational technologies, using whatever technologies are supported by the institution where they are enrolled (Henderson et al., 2015). Program design, assessment modes, instructional approaches and educational resources influence students’ ways of working. Institutional learning management systems present students with online learning materials that supplement or replace face-to-face learning and teaching, and by default, direct technology-enabled learning experiences and approaches for students (Henderson et al., 2015).

Learning technologies are linked with controversial rhetoric in academic and topical literature. Phrases like *technology-enhanced learning*, *connected learning* and *flipped classrooms* do not fully substantiate claims of technology facilitating learning (Selwyn, 2016). Communications technologies, virtual classrooms and learning management systems extend beyond physical boundaries, potentially giving students asynchronous access to learning activities.

As non-traditional students’ technological preferences, personal demographic characteristics and self-regulation are expected to predict their ASE in a BL context and have pedagogical implications, we address the following research questions.

*RQ1.* What technological tools do students use to support their learning?

*RQ2.* What predictors influence non-traditional students’ self-efficacy and confidence to complete their studies?

*RQ3.* What are the pedagogical implications for designing BL experiences?

## Method

### Setting and participants

Respondents in this pre-COVID study were undergraduate students from four campuses of a fast-growing, regional Australian university using BL. This study employed a descriptive research design using survey data and formed part of a larger research project on BL across several disciplines. Blended learning at this university is generally used to support predominantly face-to-face teaching. The learning management system provides a structure within which BL is deployed, based on institutional standards. A range of technological tools are used to enrich students’ learning experiences, although design elements vary, depending on disciplines and unit learning outcomes. The participants were enrolled in Business, Creative Industries, Social Sciences, Natural Sciences, Health and Engineering programs. Students were invited to complete the survey using a promotional video embedded in student emails. The response rate was 9.2%. While 470 students completed the survey, only 204 responses were usable due to the number of incompletes and/or indecipherable responses submitted. Among the student sample 75.4% identified as female, while 39.7% of students indicated they were first-in-family. The majority (73.5%) were enrolled in full-time study and 32.4% of respondents were first year students, 29.4% were in second year and 31.9% were in their final year of study. Most respondents (59%) self-reported they generally achieved a distinction (75% and higher) in their units, indicating the sample skewed towards higher-achieving students. More than three quarters (76%) of students were 22 years and older, indicating most respondents were mature-aged students. The sample is diverse and is representative of the non-traditional undergraduate student cohort at this regional university. The student population has a median age of 26 years, with 72% studying full-time, the majority are female (61%) and nearly half of students being first in family [deidentified institution website, 2021]. Small sample sizes are suitable to make inferences from, provided it accounts for the degree of the diversity in the population on key variables (Vaus, 2001).

### The survey

*Participants*. With ethics approval granted, the survey was pre-tested with a student reference group (n = 17; 11 females, 6 males) and reviewed for comprehension, clarity, and relevant language. The student reference group members worked through the survey individually, then formed small groups to discuss thoughts and concerns, and finally communicated back to the full group. The amended survey was administered online during Weeks 8–13 of Semester 2, 2018. It was promoted to all undergraduate students by formal *Student Communication* emails in Week 8, with reminder emails in Weeks 11 and 13 and informally promoted by the student reference group and project team members. Formal email communications contained a recruitment video featuring student reference group members. Morning tea and $20 gift card incentives were offered to student reference group members.

### Measures

*Demographics.* Students reported their age (in years), gender (male, female, other), study load (full-time, part-time), if anyone in their family had previously attended university (as “parents/siblings”, “extended family”, or “I am the first to go to university”. Students were also asked what grade level they perceived that they “usually achieved”, as “I would not like to say”, Fail, Pass, Credit, Distinction, or High Distinction, and which were coded as the letter grades of the university, i.e., Pass (4), Credit (5), Distinction (6), High Distinction (7) with Fail (3) and “not say” (1).

*Personal characteristics and preferences for technology use*. The survey measured *self-regulated learning* (SRL) (Zimmerman et al., 1992) with 11 items that asked how well students felt they could perform study activities (sample item., “finish assignments by deadlines”), rated from 1, not at all well to 5, very well. Students’ use and confidence with technologies was assessed as the *perceived suitability* of the available BL technology the students used (3 items, sample item, “technologies I use help me engage in course material”), their *future career confidence* of the technologies they use (1 item, “technologies help to prepare me well for the workplace”) and their *technological independence* using technologies not included in their degree program, but as part of their chosen career field (2 items, sample item “I use technologies different to those utilised in my courses”). These items were rated on a 5-point scale (1, strongly disagree to 5, strongly agree).

*Perceptions and use of BL technologies at university.* First, students’ use and perceived benefits of the university’s learning and teaching technologies, e.g., lecture recordings, video resources, online/cloud collaboration, were assessed. The ten technologies were rated from 0 (have not used this), 1, not all beneficial to 7, most beneficial. For each technology, students were then grouped as “did not use technology” (0), “not beneficial” (rated as 1–3), “neutral” (rated as 4), or “beneficial” (rated as 5–7). Secondly, students reported how often they collaborated with other students (e.g., email, Google Docs, Facebook messenger) and then how often they interacted with their lecturers (e.g., email, discussion boards). Frequency of use via each of the different tools was assessed on a 5-point scale, from 1 never to 5 always.

*Outcomes*. The outcomes for the survey were measured as academic self-efficacy (ASE) and perceived academic achievement. ASE (Peterson & Whiteman, 2007) was measured with three items (rated from 1, not at all to 5, very certain; Peterson & Whiteman 2007). Students reported how confident they felt they were in completing first, the current semester in which they were enrolled, and second, all the requirements to finish their degree on a scale of 0, not at all confident, to 10, very confident.

### Data analysis

Once the data had been collected, it was downloaded into SPSS for analysis. Scales were summed and then averaged for use. Cronbach’s alphas are given in Table 2. First, the use of the various technologies was assessed as whether the technology was beneficial or not and how frequently different means of collaboration were used. Second, ANOVAs compared students based on their ages and whether they were “first in family” for each of the outcomes (ASE, confidence to complete the semester and the degree). Third, the predictors of the outcomes were assessed. As students can overestimate their abilities on academic tasks (e.g., Kruger & Dunning 1999; Serra & DeMarree, 2016), the distributions of confidence ratings were undertaken. As these ratings were strongly negatively skewed and therefore, non-normally distributed, both confidence questions were dichotomised into “somewhat confident” (0 to 8) and “very confident” (9–10) to enable analysis. Analyses were then completed with Hierarchical Multiple Regression for ASE, and binary logistic regression used for both confidence questions.

## Results

### Descriptive statistics

#### Technological tools students use to support their learning

Students were asked to indicate which technological tools support their learning, first focusing on their individual current study experiences, when collaborating with other students on group assessments, and supporting interactions with faculty (referring to lecturers, tutors and/or unit coordinators).

Table 1 shows the technological tools students found most beneficial to support their learning, with the most beneficial being lecture recordings then video resources. More than two-thirds of students (71.9%) indicated lecture recordings were beneficial, 57.8% also identified video resources beneficial, whilst more than a half (55.9%) indicated social media to be useful. This perceived value and benefit correlates with the frequency of use across the cohort. A large group of students indicated they had not experienced or utilised gamification (86%) or simulation (70.9%), as technological tools. Less than half of the sample were familiar with polling tools and e-portfolio. Of these, 43.2% did not find polling useful, while 53% did not find e-portfolios beneficial.

**Table 1:** Different types of BL technologies available for students, and their use and perceived benefits, arranged from most to least used

	Students who did not use this technology N	Students’ use and perceived benefits of each technology					
Type of BL technology		N	Mean	SD	Not beneficial (Rated 1 to 3) %	Neutral (Rated 4) %	Beneficial (Rated 5 to 7) %
Lecture recordings	11	192	5.35	1.69	15.1	13.0	71.9
Video resources	39	161	4.78	1.72	24.2	18.0	57.8
Social media	63	136	4.44	1.98	32.4	11.7	55.9
Online meetings	101	100	4.44	1.66	30.0	18.0	52.0
Cloud collaboration	104	98	3.92	2.08	43.9	12.2	43.9
E-portfolios	104	100	3.30	2.07	53.0	15.0	32.0
Polling tools	111	88	3.72	1.67	43.2	22.7	34.1
Content production and editing tools	123	74	4.73	1.72	23.0	17.6	59.4
Simulation	144	59	4.64	1.79	30.5	11.9	57.6
Gamification / Online games	172	28	4.04	2.06	42.9	10.7	46.4

Students (N = 204) reported how frequently they used a range of technological tools when collaborating with other students to study together or work on group assessments (rated as 1, never to 5, always). The tools used most often were email (M = 3.64, SD = 1.22), Facebook Messenger (M = 3.45, SD = 1.41) and Google Docs (M = 2.11, SD = 1.28), respectively. Most students indicated they never used other messaging applications, such as WhatsApp (91%) or video conferencing tools such as Skype (90.5%), Zoom (94.7%) or Google Hangouts (92.5%) for collaboration. Finally, students (N = 204) identified the technological tools they used to interact with faculty (rated from 1, never to 5, always) with email being the most common (M = 4.16, SD = 0.93), followed by Blackboard’s discussion board (M = 2.26, SD = 1.18) and unit Facebook groups (M = 1.64, SD = 1.14).

#### ANOVAs for non-traditional students based on their status of first-in family and their age group

Before commencing the regression analyses, students were compared on their average grades, self-regulated learning, academic self-efficacy, and confidence to finish their current units and degrees. First-in-family was grouped as parents and/siblings (n = 86), extended family (n = 35), or ‘I am first in family’ (n = 79). There were age differences for these groups, (F(2,197) = 3.11, *p* = .047) with students who were first in family (M = 33.9, SD = 12.8) being slightly older (M = 31.8, SD = 12.3, t(163) = 2.49, *p* = .041). The groups did not differ on how they viewed technology, their average grade or self-regulated learning, their academic self-efficacy, or their confidence to complete their current units or degrees. The lack of differences here suggests that for this sample being first-in-family was not a barrier to academic success.

Ages were grouped as 17–21 (24.0%), 22–35 (32.5%), 35–49 (26.0%) and over 50 years (17.5%). The ANOVAs found no differences in age groups for average grades or self-regulated learning, or confidence to complete current units and degrees. There were slight age differences for academic self-efficacy (F(3,196) = 2.79, *p* = .042), with 22–35 year age group (M = 10.65, SD = 2.57) being slightly less confident than the 17–21 age group (M = 11.85, SD = 2.11), t(111) = 2.61, *p* = .057 (with Bonferroni adjustments). Being an older student was also not a barrier to academic success.

### Predictors of ASE and confidence to complete

#### Bivariate correlations

The means, SD, and correlations between variables are shown in Table 2. Examining what supports students’ perceived success in their learning experiences, the correlations suggest significant associations between factors. In terms of academic self-efficacy and self-reported unit completion, there were significant positive correlations between the outcomes. These results correlated with self-regulated learning (SRL), average grade, and the perceived suitability of the available educational technologies. Students’ age and gender, how beneficial they found the lecture recordings and video resources, and how often they contacted their unit staff were not significantly correlated with the outcomes, although emailing peers was significantly and positive correlated with academic self-efficacy in a BL context.

**Table 2:** Means. Standard deviations and correlations between the variables (N=197)

	Mean	SD			1		2	3			4		5	6		7	
1 Age	33.69	13.13			-		-0.04	0.12†			0.11		-0.01	-0.30***		-0.08	
2 Gender	1.76	0.43					-	0.08			0.05		-0.03	0.02		0.12†	
3 SRL	3.76	0.65						(0.855)			0.34**		0.09	0.24***		0.05	
4 Average grade	5.42	1.39									-		0.15*	0.26***		0.14*	
5 Year level	2.86	1.18											-	-0.02		-0.11	
6 Perceived suitability of technology	3.78	0.79												(0.676)		0.46***	
7 Future career confidence of tech	3.52	1.01														-	
8 Technological independence	2.94	0.88															
9 Lecture recordings	5.04	2.05															
10 Video resources	3.85	2.43															
11 Facebook Messenger	3.48	1.41															
12 Email to students	3.63	1.22															
13 Email to staff	4.16	0.94															
14 Academic SE	3.78	0.82															
15 Complete this semester	9.03	1.92															
16 Complete degree	8.73	2.14															
	8		9	10		11			12	13		14			15		16
1 Age	-0.15*		0.12†	-0.09		-0.31***			0.09	-0.24**		0.01			-0.04		-0.11
2 Gender	-0.14*		0.21**	0.18**		0.15*			-0.01	0.03		-0.12†			-0.06		-0.04
3 SRL	-0.10		-0.04	0.01		0.06			0.11	0.09		0.55***			0.32***		0.41***
4 Average grade	-0.12		-0.01	0.03		0.10			0.03	0.04		0.23**			0.21**		0.21**
5 Year level	-0.08		0.00	-0.15*		0.12			0.13†	0.13†		-0.06			-0.04		0.04
6 Perceived suitability of technology	-0.09		0.07	0.19**		0.16*			0.10	0.24**		0.31***			0.24**		0.36***
7 Future career confidence of tech	-0.40***		0.10	0.09		0.05			0.11	0.08		0.08			0.11		0.19**
8 Technological independence	(0.608)		-0.13	0.06		0.04			-0.01	0.06		-0.01			-0.14*		-0.14†
9 Lecture recordings			-	0.28***		-0.01			0.15*	0.09		0.01			0.00		0.04
10 Video resources				-		0.06			0.10	0.12		0.07			-0.04		0.03
11 Facebook Messenger						-			0.14*	0.27***		0.04			0.02		0.10
12 Email to students									-	0.25***		0.20**			0.05		0.07
13 Email to staff										-		0.12†			-0.02		0.03
14 Academic SE												(0.862)			0.37***		0.47***
15 Complete this semester															-		0.74***
16 Complete degree																	-

Note. † *p* < .10, * *p* < .05, ** *p* < .01, *** *p* < .001. Cronbach’s alpha on diagonal in brackets, single items indicated by ‘-’

#### Hierarchical regression analyses

Hierarchical multiple regressions (HMRs) were used to determine how students’ characteristics, self-regulated learning, perceived technological competence and student-rated beneficial technologies predicted three outcomes: academic self-efficacy, confidence to complete the current semester, and self-reported confidence to complete the degree.

Variables were entered as blocks, with Block 1, the characteristics of the student (i.e., demographics, self-regulated learning, average grade, year of degree), Block 2, their experience with the educational technologies (i.e., perceived suitability, future career confidence with technologies, and technological independence), and Block 3 (i.e., beneficial experience of technology, interacting with other students and staff).

Assumption testing for the regressions showed these were not breached for academic self-efficacy and results of the HMR are shown in Table 3. Only the first block added significantly to the explanation of academic self-efficacy in a BL context and the final model explained substantial variance, Adj R^2^ = 0.34, *F*(13,177) = 8.49, *p* < .001. By Cohen’s (1992) conventions, this is a very large effect (*f*^2^ = 0.66). Self-regulated learning (SRL) was the strongest predictor for academic self-efficacy, as greater SRL is positively linked to greater academic self-efficacy. Gender and year of degree as significant predictors showed evidence of suppression (i.e., should be interpreted with caution) and perceived suitability of technology marginally significant.

**Table 3:** Hierarchical multiple regression with academic self-efficacy as outcome (n = 191)

		Step 1	Step 2	Step 3
	ΔR^2^	β	β	β
Block 1	0.345***			
Age (in years)		− 0.06	− 0.01	− 0.04
Gender		− 0.16**	− 0.15*	− 0.16*
SRL		0.56***	0.53***	0.52***
Average grade achieved, e.g.,7 = HD		0.05	0.02	0.04
Year of degree		− 0.11†	− 0.10†	− 0.13*
Block 2	0.016			
Perceived suitability of technology			0.15*	0.13†
Future career confidence of tech			− 0.02	− 0.04
Technological independence			0.02	0.01
Block 3	0.024			
Lecture recordings are beneficial				0.05
Video resources are beneficial				0.02
Use Facebook Messenger to contact peers				− 0.01
Email to students				0.13
Contact staff with Email				0.03
Adj R^2^ of final model	0.339***			

Note. † *p* < .10, * *p* < .05, ** *p* < .01, *** *p* < .001

Assumption testing for confidence to complete current semester and their degrees showed scores were significantly negatively skewed and highly kurtotic, with most of the sample being very confident both outcomes would occur (55.4% of the sample were completely confident). The regressions for this outcome were not completed due to these variations and instead, binary logistic regression was used to assess which of the variables would increase or decrease the likelihood students would be very confident of completion. Confidence for both items was categorised as ‘somewhat confident’ (0, ratings of 0 to 8) or ‘very confident’ (1, ratings of 9 or 10) and the same variables as the HMR were entered as predictors in the logistic regressions. As shown in Table 4, the models were significant (i.e., omnibus test) and explained large variance, and were a good fit of the data (i.e., Hosmer and Lemeshow test was non-significant).

**Table 4:** Summary of fit indices for the binary regression models for completion of current semester and the degree (N = 191)

Tests of model fit	Complete current semester	Complete degree
Omnibus test for model	X^2^(13) = 37.13, p < .001	X^2^(13) = 46.22, p < .001
Snell and Cox R^2^	0.177	0.215
Nagelkerke R^2^	0.273	0.315
Hosmer and Lemeshow Test	X^2^(8) = 12.49, p = .131	X^2^ (8) = 3.59, p = .862
Cases correctly categorised	80.1%	78.5%

The results of the binary logistic regressions are shown in Table 5, with focus on the odds ratio reported as Exp(B). Of the predictors, self-regulated learning was a highly significant predictor, strongly increasing the likelihood of students reporting feeling very confident. When self-regulated learning increased by one unit (on scale of 1–5), there was a substantial increase in the probability that students being in the ‘very confident’ group: 3.46 times (i.e., 346%) more confident for completing the current semester and 5.17 times (i.e. 517%) more confident for completing their degree. Students who found the technology suitable for the unit’s purpose approached significance to increase confidence for both semester and degree (although the confidence intervals include 1.00). Interestingly, feeling very confident of completing the current semester was almost halved where the student had more frequent email contact with academics (i.e., 42% less likely) and by being technologically independent (i.e., 40% less likely), although this latter was approaching significance (confidence interval includes 1.00).

**Table 5:** Predictor coefficients for the models that predict students’ confidence to complete the current semester and complete their degree (N = 191)

	Complete current semester				Complete degree		
	B	SE(B)	Exp(B) [95% CI]		B	SE(B)	Exp(B) [95% CI]
Constant	-1.08				-5.37		
Age (in years)	-0.01	0.02	0.99 [0.95, 1.03]		-0.02	0.02	0.98 [0.95, 1.02]
Gender	0.09	0.48	1.09 [0.42, 2.80]		-0.21	0.47	0.81 [0.33, 2.03]
SRL	1.24	0.37	3.46**[1.69, 7.10]		1.64	0.38	5.17*** [2.46, 10.85]
Average grade	0.15	0.14	1.16 [0.88, 1.53]		0.04	0.14	1.04 [0.79, 1.36]
Year of enrolment	-0.07	0.18	0.94 [0.66, 1.33]		0.25	0.17	1.29 [0.92, 1.80]
Perceived suitability of technology	0.54	0.34	1.72† [0.89, 3.32]		0.51	0.31	1.66† [0.90, 3.05]
Future career confidence of tech	-0.13	0.25	0.88 [0.54, 1.44]		0.03	0.24	1.03 [0.64, 1.64]
Technological independence	-0.51	0.27	0.60† [0.35, 1.02]		-0.06	0.25	0.94 [0.57, 1.55]
Lecture recordings	-0.01	0.11	0.99 [0.79, 1.24]		0.07	0.10	1.07 [0.87, 1.31]
Video resources	-0.07	0.09	0.94 [0.78, 1.13]		-0.07	0.09	0.93 [0.78, 1.11]
FB Messenger	-0.02	0.16	0.99 [0.72, 1.34]		0.15	0.14	1.16 [0.87, 1.53]
Email other students	0.04	0.18	1.04 [0.73, 1.49]		-0.09	0.18	0.91 [0.64, 1.29]
Email to staff	-0.55	0.26	0.58* [0.35, 0.96]		-0.35	0.23	0.71 [0.45, 1.10]

Note. † p < .10, * p <. 05, ** p < .01, *** p < .001.

## Discussion

The findings from our study suggest the importance of self-regulated learning for non-traditional students’ perceived success. Our results propose educators should consider student circumstances, and their beliefs about their ability to perform. Concepts such as academic self-efficacy and self-regulated learning are useful as they play a critical part in the learning process (Guo et al., 2019). Student characteristics and preferences for digital technologies also play a supporting role in their learning. Our findings propose the importance of being acquainted with non-traditional students’ “lived reality, not just institutional and/or systemic interests*”* (Mackaskill & Denovan, 2013, p. 747). Our results suggest educators should consult regularly with students to determine their familiarity with technological tools to ensure BL technologies suit students’ preferences and industry needs, whilst being academically fit-for-purpose.

The technological tools non-traditional students perceived as beneficial to learning were lecture recordings and video resources, while simple tools such as email and Facebook Messenger extended communication. Students’ expectations included direct instruction via lecture recordings and videos, coupled with opportunities to communicate via email, discussion board and online chat. Students who experienced educational games and simulations rated them less beneficial than direct instruction and communication applications. Students regarded e-portfolios as marginally unbeneficial. These findings align to what Henderson et al., (2017, p. 1576) called “structured reviewing” of learning materials. Students prefer explicit clarifications *of* knowledge and have little tolerance for ambiguity or open-ended approaches designed to engage them *with* knowledge. Students reported being satisfied with online materials that support flexible autonomous study and deliver what is required; anything more can be deemed superfluous.

Student engagement with learning approaches represents learning behaviour. Learning behaviour is one of three contributors to self-regulated learning, the others being metacognition and motivation (Zimmerman & Martinez-Pons, 1988). Metacognition encapsulates qualities of personal organisation and self-evaluation that support learning autonomy. Motivation is akin to academic self-efficacy, itself a contributor to academic success and measured by program completion, level of achievement and satisfaction (Honicke & Broadbent, 2016; Puzzifero, 2008). In our study, academic self-efficacy and capacity for self-regulated online learning contributed to perceived positive graduation pathways in blended programs, regardless of students’ prior socio-economic status. This was reinforced by our finding that students who most frequently emailed their instructors for support tended to be the least confident about their perceived ability to achieve academic success. Similar to research by Puzzifero (2008), there were indications that students who make frequent contact (showing higher levels of help-seeking) do so because they perceive assessment tasks to be ambiguous and because they lack ASE.

ASE increased where students found the university’s educational technologies sufficient for their learning needs, and waned when students migrated to technologies outside those provided by the university. The negative correlation between ASE and technological independence only approached a possible significance, this represents an area for further research. Nevertheless, the results suggest students perceive they benefit more when instructors present them with purposeful technologies rather than leaving students to make selections without guidance. Our results suggest, a quality BL environment will facilitate self-regulated learning, using endorsed technological applications.

Our combined findings about non-traditional student perceptions and preferences for BL emphasise the relationship between student capacity for self-regulated learning and students’ self-efficacy in blended programs. Contrasting the student perspective, Panadero (2017) found instructors in higher education settings focused more on unit content, paying little attention to facilitating self-regulated learning among students. Based on Panadero’s (2017) review and our findings, we recommend these instructors should support student self-regulated online learning strategies to support successful outcomes.

Our results suggested a positive correlation between student self-efficacy, and confidence to complete their studies and the perceived suitability of technology. However, our non-traditional student sample are self-reported high achievers and not a comprehensive representation of learner demographics. Whilst increased flexibility and autonomy associated with BL are beneficial for learners who possess self-regulated learning skills, without guidance and support this can challenge the learning of low achievers (Owston et al., 2013). Curriculum design and selection of technological tools should be guided by self-regulated learning theory to develop and nurture academic self-efficacy and self-regulation qualities. When designing online learning activities and choosing appropriate technology, it is important to consider regular checkpoints for students to self-evaluate not only their mastery of content, also to assess their learning process (Guo et al., 2019). This is an opportunity for educators to monitor progress and check for misconceptions. Monitoring student progress can provide insights into individual workloads and student capabilities. In some cases, intervention and additional learner support may be needed in order for the student to meet the learning outcome and to motivate learner confidence (Zimmerman, 1995). The choice and application of technologies should be relevant to students’ academic development and authentic to professional practice. The purpose and intent of learning technologies should be clearly communicated and explicitly aligned with the intended learning outcomes. Students who understand the purpose of specific technological tools in their learning context are more inclined to persist even when the experience is challenging.

Whilst lecturers have little control over the intrinsic factors that motivate a students’ interest and commitment to a subject, an educator’s attitude can influence student experience and engagement (Guo et al., 2019). Providing ‘how-to’ resources and encouragement can help students overcome digital anxiety and improve self-efficacy with learning technologies. Involving students as partners in the learning process can enhance their sense of agency and positively affect self-regulation and motivation. This can increase students’ sense of belonging in the e-learning community and foster positive attitudes toward BL.

This study is not without its limitations. The sample of this cross-sectional study of students from one institution cannot infer causality from the findings. Additionally, the sample size was limited by the number of incompletes and/or indecipherable responses received. Students were mostly high-achieving and expressed their perceived confidence that they would finish the semester, which may limit generalisability to students with different motivation levels. Achievement questions were self-reported which could be problematic as students tend to overestimate their abilities on academic tasks. Self-reported data can pose reliability and validity limitations; therefore, any correlations could potentially be explained by other factors not controlled for in this study. We aimed to s address by dichotomising these questions, as explained in the methods section, but this still poses a significant limitation, nonetheless. The scales for technology use had Cronbach’s alphas at the lower end of acceptable values. These associations should be investigated further in future research in addition to the impact of peer-to-peer support in self-regulation and academic self-efficacy.

## Conclusion

In an attempt to garner non-traditional students’ views of BL tools, this study investigated the perceptions of 204 undergraduate students regarding their preferred BL approaches, academic self-efficacy and self-regulated learning. Contrary to the ever-growing selection of learning technologies available, results show students prefer lecture recordings and video resources to support their learning, and email and Facebook Messenger to communicate with peers and academic staff. Our study suggests that quality BL environments are consistent, relevant, and effective, while facilitating self-regulated learning using university endorsed and purposeful technological applications. Additionally, our findings propose academic self-efficacy can increase if students perceive the educational technologies supplied by their institution are sufficient for their learning needs. These findings offer a guide for educators about ways to adapt to the new digital norm of the post-COVID world. Creating supportive online learning environments utilising fit-for-purpose technologies which nurture self-regulated learning, develop communication skills, and embrace professional qualities and attributes will be increasingly important. Understanding the experiences and expectations of the different learner cohorts is central to these processes.
